# Real-World Efficacy of Ensitrelvir in Hospitalized Patients With COVID-19 in Japan: A Retrospective Observational Study

**DOI:** 10.7759/cureus.61048

**Published:** 2024-05-25

**Authors:** Ryohei Yoshida, Takaaki Sasaki, Yoshinobu Ohsaki

**Affiliations:** 1 Institute of Biomedical Research, Yoshida Hospital, Asahikawa, JPN; 2 Department of Internal Medicine, Division of Respiratory Medicine and Neurology, Asahikawa Medical University, Asahikawa, JPN; 3 Department of Internal Medicine, Division of Respiratory Medicine and Neurology, Asahikawa Medical University Hospital, Asahikawa, JPN

**Keywords:** hospitalization, ensitrelvir, covid-19, antiviral medications, antigen negative conversion

## Abstract

Background and aim: The coronavirus disease 2019 (COVID-19) pandemic necessitates continuously evaluating antiviral treatments, especially for high-risk groups, including older individuals. This study aimed to compare the efficacy of three antiviral drugs, including remdesivir, molnupiravir, and ensitrelvir, in hospitalized patients as measured by our own institution's antigen test, focusing on outcomes, such as severe acute respiratory syndrome coronavirus 2 (SARS-CoV-2) antigen levels, hospitalization duration, and fever resolution.

Methods: This retrospective observational study was conducted at Yoshida Hospital, Asahikawa City, Japan, enrolling 154 patients who received antiviral treatment upon COVID-19 diagnosis from July 1, 2022, to September 15, 2023. The diagnosis was confirmed by proprietary antigen tests or loop-mediated isothermal amplification assays. Patients who received treatment outside the hospital or with consistently negative antigen results were excluded. Drug administration was determined by attending physicians, considering oral administration challenges and renal dysfunction. The data were statistically analyzed using an unpaired two-tailed Student’s t-test and one-way analysis of variance complemented by the Tukey post-hoc test for detailed group comparisons.

Results: No significant differences were observed in the initial antigen levels among the treatment groups. By day 10, the ensitrelvir group showed lower antigen levels than the other groups, but not significantly. The ensitrelvir group had a higher antigen-negative conversion rate and a significantly shorter hospital stay than the molnupiravir group. However, no significant differences were noted in the fever resolution time among the groups.

Conclusion: This study suggests the potential benefits of ensitrelvir in reducing antigen levels and hospitalization duration. However, the overall efficacy of the antiviral agents for symptomatic relief appears similar. These findings underscore the need for further research to optimize COVID-19 management by considering personalized treatment approaches and long-term outcomes.

## Introduction

The novel coronavirus classification in Japan has shifted from Category 2 to Category 5 under the Infectious Disease Control Law as of May 2023. This classification signifies the persistent necessity to address treatments for coronavirus infections. The development of vaccines against COVID-19 has contributed significantly to the prevention of the disease and mitigation of its severity [1,2]. However, multiple factors, including advanced age, diabetes, and hypertension, reportedly contribute to an increased risk of severe disease [3-5]. Therefore, treatments, including those involving antiviral medications, play a critical role in clinical management.

Several antiviral drugs, including remdesivir, molnupiravir, nirmatrelvir/ritonavir, and ensitrelvir, are available for clinical use in Japan. Consequently, the selection of suitable antiviral medications after hospitalization is critical. Several studies have assessed the efficacy of various antiviral drugs when treating COVID-19 [6,7]. However, research comparing the effectiveness of distinct antiviral medications, including ensitrelvir, in hospitalized patients is scarce. Using real-world data from electronic health records and patient follow-ups, we aimed to compare the efficacy of three different drugs administered post-hospitalization in patients who tested positive for COVID-19 and required hospitalization at our institution, specifically measuring outcomes, such as the SARS-CoV-2 antigen levels, hospitalization duration, and fever resolution. This article was previously posted to the Research Square preprint server on Feb 6, 2024.

## Materials and methods

Study design

This retrospective observational study enrolled 154 patients who received antiviral treatment upon admission to Yoshida Hospital, Asahikawa City, Japan, from July 1, 2022, to September 15, 2023, who received one of the specified antiviral treatments (remdesivir, molnupiravir, or ensitrelvir) upon admission. COVID-19 diagnoses were confirmed using either the institution's proprietary antigen test or the loop-mediated isothermal amplification (LAMP) assay. We used HISCL SARS-CoV2-Ag assay kit (sysmex Cat.#CP301035; Sysmex, Germany) as our coronavirus antigen qualitative kit. A negative result was an antigen level of < 1.0 pg/ml.

Exclusion criteria were as follows: (1) Patients who were not hospitalized, (2) Patients whose antigen test results on the 10th day post-diagnosis were not confirmed, (3) Patients who did not receive any of the specified antiviral treatments during their hospital stay, and (4) Patients who tested positive via LAMP but consistently displayed negative SARS-CoV-2 antigen results, with an antigen level of <1.0 pg/ml.

The attending physician determined the choice of drugs administered. Remdesivir is often administered to patients who are at a higher risk of aspiration pneumonia or who have swallowing difficulties. Contraindications and renal dysfunction were considered when prescribing the medications. To ensure the accuracy of administering medications for patients with potential contraindications and renal dysfunction, our hospital has implemented a tightly coordinated protocol with the pharmacy department. This protocol ensures the absence of contraindications before prescribing ensitrelvir, and that renal function is assessed to ensure that patients with severe renal dysfunction (eGFR <30 mL/min) are not dosed. This study was approved by the Institutional Review Board of Yoshida Hospital (approval no. 20230809003). Informed consent was obtained in the form of an opt-out on the website. This study was performed in accordance with the Declaration of Helsinki. 

Data collection

The following patient data were obtained from electronic case records: age, sex, body mass index, comorbidities (underlying condition, cardiovascular diseases including hypertension, diabetes mellitus, dyslipidemia, chronic kidney disease, chronic liver disease, chronic obstructive pulmonary disease (COPD), cancer, dementia) hospitalization in the past year, vaccination history. We also obtained data on the number of days in the hospital stay, fever as a clinical symptom during the course of infection, and medication (remdesivir, molnupiravir, and ensitrelvir). If a record was missing in the patient data, it was categorized as 'unknown'. Patient records were accessed through the electronic health record system, which includes comprehensive clinical data for all admitted patients. One trained research assistant independently extracted data for this study. Discrepancies were resolved through discussion with a senior researcher to ensure data accuracy.

Statistical analyses

Statistical significance was assessed using an unpaired two-tailed Student’s t-test and one-way analysis of variance, followed by the Tukey post-hoc test. A p-value of <0.05 was considered statistically significant. Asterisks indicate significance, with * indicating p<0.05 and ** representing p<0.005. Columns represent means ± standard errors of the mean (SEMs). GraphPad Prism7 (GraphPad Software Inc., San Diego) was used for all statistical analyses.

Data availability 

The datasets generated during and/or analyzed in this study are available from the corresponding author upon reasonable request.

## Results

Verification of SARS-CoV-2-antigen levels

We conducted a comparative analysis to validate the antigen levels of hospitalized patients when they were administered various drugs (Table 1).

**Table 1:** Clinical characteristics of COVID-19 patients Data are represented as the number of patients (%). COVID-19: Coronavirus disease 2019; COPD: Chronic obstructive pulmonary disease

	Remdesivir	Molnupiravir	Ensitrelvir
Variables	(N=85)	(N=44)	(N=25)
Age at admission			
50s	1(1.2)	0(0)	1(4)
60s	3(3.5)	4(9.1)	3(12)
70s	12(14.1)	11(25)	7(28)
80s	33(38.8)	22(50)	10(40)
90s	33(38.8)	7(15.9)	4(16)
100s	3(3.5)	0(0)	0(0)
Sex			
male	29(34.1)	25(56.8)	11(44)
female	55(64.7)	19(43.2)	14(56)
Underlying conditon			
Cardiovascular diseases (Including hypertension)	53(62.3)	33(75)	17(68)
				diabetes mellitus	30(35.3)	18(40.9)	10(40)
Dyslipidemia	22(25.9)	6(13.6)	4(16)
Chronic kidney disease	20(23.5)	24(54.5)	2(8)
Chronic liver disease	0(0)	2(4.5)	2(8)
COPD	7(8.2)	1(2.3)	4(16)
Cancer	13(15.3)	11(25)	3(12)
Dementia	39(45.9)	10(22.7)	6(24)
Depression/Schizophrenia	3(3.5)	3(6.8)	0(0)
Stroke	12(14.1)	8(18.2)	7(28)
Body mass index			
<25	81(95.3)	40(90.9)	22(88)
25-29	3(3.5)	3(6.8)	2(8)
≧30	1(1.2)	1(2.3)	1(4)
Hospitalization in the past year			
Yes	35(41.2)	29(65.9)	15(60)
No	50(58.8)	15(34.1)	10(40)
vaccination history			
No vaccination	5(5.9)	1(2.3)	1(4)
Yes	59(69.4)	36(81.8)	22(88)
1 time	1(1.2)	1(2.3)	1(4)
2 times	7(8.2)	1(2.3)	1(4)
More than 3 times	51(60)	34(77.3)	20(80)
Unknown	21(24.7)	7(15.9)	2(8)

**Figure 1:**
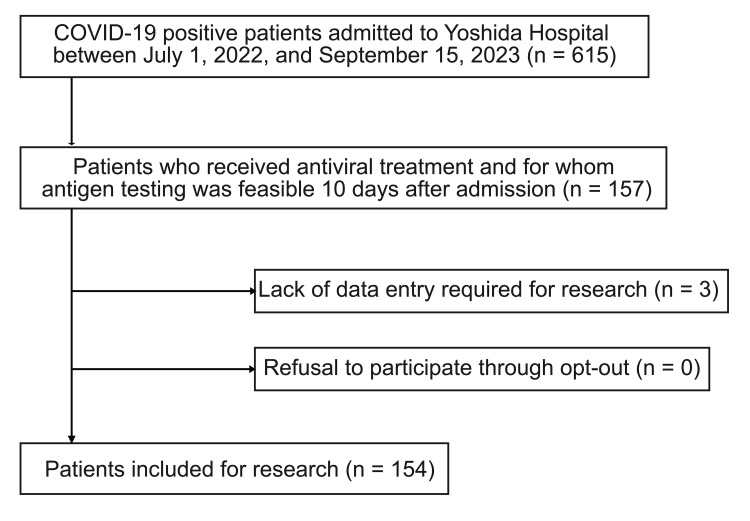
Flowchart describing patient selection All patients were hospitalized during the study period (July 1, 2022 to September 15, 2023).

The number of patients enrolled in this study was 154 (Figure 1).

**Figure 2:**
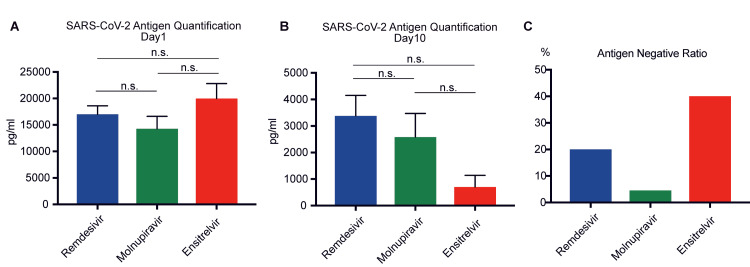
Comparison of SARS-CoV-2 antigens (a) Antigen levels on day 1 of hospitalization. (b) Antigen levels on day 10 after each antiviral treatment. (c) Antigen negative ratio for each antiviral treatment. Statistical significance was assessed using an unpaired two-tailed Student’s t-test and one-way analysis of variance, followed by the Tukey post-hoc test. A p-value of ≧0.05 was not significant (n.s.). Error bars represent means ± standard errors of the mean (SEMs). SARS-CoV-2: Severe acute respiratory syndrome coronavirus 2

The patient cohort comprised 88 women and 66 men. The average age was notably high, at 83.7 (range 53-104) years, owing to the large number of patients admitted to our hospital from chronic care facilities and nursing homes. Upon quantitatively assessing the SARS-CoV-2 antigen levels at admission, no discernible differences in antigen concentrations across the treatment (remdesivir (17007±1599 pg/ml), molnupiravir (14291±2315 pg/ml), and ensitrelvir (19996±2808 pg/ml)) groups were noted (Figure 2a).

**Figure 3:**
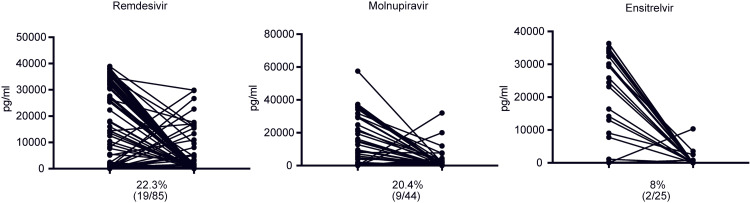
Comparison of day 1 and 10 antigen resurgence rates among individuals for each antiviral treatment

Upon evaluating the antigen levels on day 10 of treatment, we observed no significant difference between the remdesivir (3383±770.7 pg/ml) and molnupiravir (2587±884.4 pg/ml) groups (p=0.5240). While the antigen levels were observed to be lower in the ensitrelvir group compared to those of the other groups, the differences did not reach statistical significance when compared to the molnupiravir (p=0.1275) and remdesivir groups (p=0.0662) (Figure 2b). Subsequently, we assessed the drug-specific SARS-CoV-2 antigen-negative conversion rate after 10 days of treatment. The ensitrelvir group had a rate of 40% (10/25), surpassing the rates of 20% (17/85) and 4.54% (2/44) in the remdesivir and molnupiravir groups, respectively (Figure 2c). To elucidate the nuanced changes in the antigen levels corresponding to the use of each drug, we tracked level alterations upon admission and on day 10 of treatment. Our findings indicated that the antigen levels surged despite therapeutic intervention. Nonetheless, the antigen resurgence rates were 8% (2/25), 22.3% (19/85), and 20.4% (9/44) in the ensitrelvir, remdesivir, and molnupiravir groups, respectively, underscoring the distinctions across the drug treatments (Figure 3).

Verification of hospitalization days

**Figure 4:**
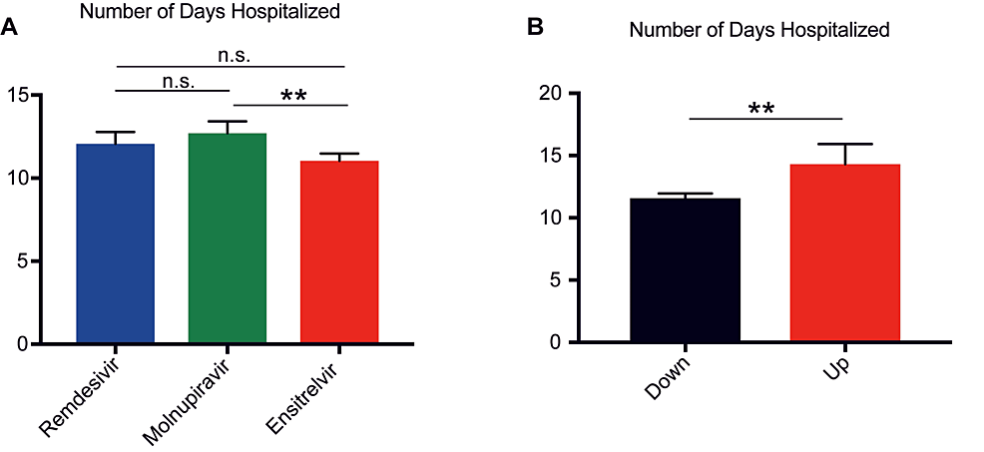
Comparison of hospitalization days (a) Hospitalization days for each antiviral treatment. (b) Comparison of hospitalization days between the up-group (Up) and down-group antigens (Down) based on Figure 2. Statistical significance was assessed using an unpaired two-tailed Student’s t-test and one-way analysis of variance, followed by the Tukey post-hoc test. A p-value of <0.05 was considered statistically significant. p-value of ≧0.05 was not significant(n.s.). Asterisks indicate significance, with ** representing p<0.005. Error bars represent means ± standard errors of the mean (SEMs).

In our subsequent analysis of hospitalization duration among the various drug groups, no significant differences were observed between the remdesivir (12.11±0.369 days) and molnupiravir (12.7±0.354 days) groups (p=0.2982) or the remdesivir and ensitrelvir (11.04±0.212 days) groups (p=0.1264). However, the ensitrelvir group demonstrated a significantly shorter hospital stay than did the molnupiravir group (p=0.0013) (Figure 4a).

Temporal changes in the antigen levels, as depicted in Figure 3, indicated differential antigen escalation rates across various drug treatments. Based on the antigen data, we classified patients on day 10 after treatment, relative to their admission day, into two distinct categories: the Down group (n=124), with decreased levels, and the Up group (n=30), with increased levels. The ensuing analysis revealed an average hospital stay of 11.57 days in the down group, compared with 14.3 days in the up group. This difference was significant, suggesting that patients in the up group tended to have longer hospitalizations than those in the down group (p<0.0001) (Figure 4b).

Time for COVID-19 fever improvement

**Figure 5:**
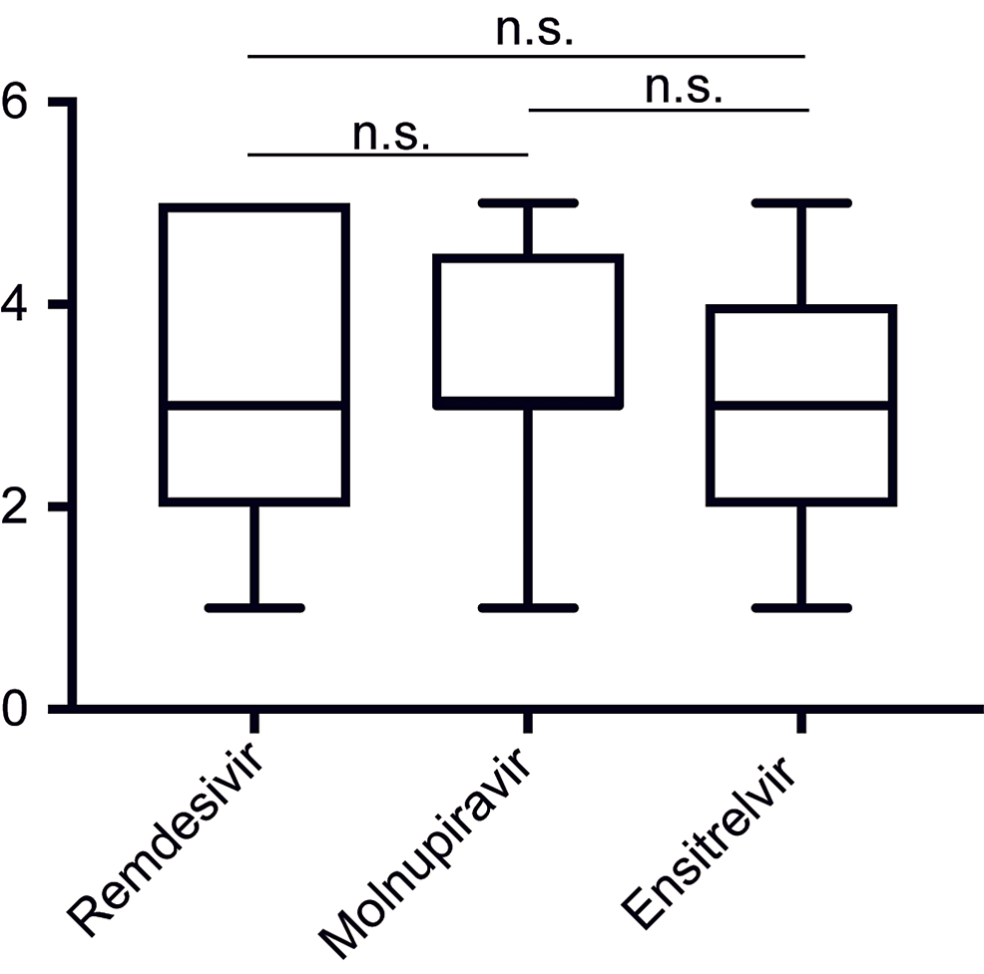
Duration before an admission temperature of >37°C decreased to <37°C with antiviral treatments Statistical significance was assessed using an unpaired two-tailed Student’s t-test and one-way analysis of variance, followed by the Tukey post-hoc test. A p-value of ≧0.05 was not significant (n.s.).

During the investigation of fever alleviation related to COVID-19, patient groups were evaluated based on the drugs they received. Upon admission, 78.8% (67/85), 80.4% (37/46), and 68% (17/25) of patients in the remdesivir, molnupiravir, and ensitrelvir groups, respectively, presented with a fever exceeding 37°C. However, analyses of the mean duration required for the fever decrease below 37°C within five days after admission revealed no significant differences between the groups (mean ± SEM days: 3.493±0.1784, 3.432±0.1958, and 2.882±0.3417 in the remdesivir, molnupiravir, and ensitrelvir groups, respectively) (Figure 5).

## Discussion

This study compared the effectiveness of remdesivir, molnupiravir, and ensitrelvir in managing hospitalized patients with COVID-19, focusing on key outcomes, including SARS-CoV-2 antigen levels, hospitalization duration, and fever resolution. Several mechanisms are currently being explored as therapeutic options for the development of antiviral agents against COVID-19. Within the scope of this study, two drugs, remdesivir and molnupiravir, function as RNA-dependent RNA polymerase (RdRp) inhibitors. Ensitrelvir is categorized as a SARS-CoV-2 main protease (Mpro) inhibitor [8]. All three drugs are currently employed in clinical settings and exhibit unique characteristics when used as antiviral medications [9-11]. Notably, oral antiviral medications are significantly advantageous as they can be self-administered to patients, enhancing the ease of treatment accessibility. This attribute is particularly beneficial for pandemic management as oral antiviral medications may play an increasingly important role in curbing the virus's spread [12].

First, our results underscore the efficacy of ensitrelvir, which is highlighted by a significantly higher antigen-negative conversion rate of 40% in the ensitrelvir group compared to 20% in the remdesivir group and 4.54% in the molnupiravir group. This indicates a more rapid viral clearance with ensitrelvir, suggesting its enhanced effectiveness in reducing the viral load, potentially controlling the spread of the virus, and alleviating the burden on healthcare systems. Additionally, although the initial antigen levels were similar across the three treatment groups, the ensitrelvir group exhibited lower antigen levels on day 10 of treatment. Although this reduction did not reach statistical significance when compared with the remdesivir and molnupiravir groups, it aligns with previous reports suggesting ensitrelvir's potential efficacy in reducing the viral load [13-15]. This observed trend underscores the need for larger trials to obtain conclusive evidence, reinforcing ensitrelvir’s potential in effectively managing COVID-19.

Second, our analysis revealed that the duration of hospital stays was significantly shorter for patients treated with ensitrelvir compared to those receiving molnupiravir, indicating that ensitrelvir facilitates shorter hospital stays and thus can reduce healthcare burdens, especially during pandemic peaks. While few significant differences were observed when compared to remdesivir, patient demographics and comorbidities may influence the duration of hospitalization [16,17].

However, it is essential to acknowledge that the efficacy of these antiviral treatments in terms of fever resolution was similar across all groups, thus indicating that while antiviral therapies, such as ensitrelvir can effectively reduce the viral load, their impact on immediate symptomatic relief, such as fever reduction, as observed in previous studies, is less pronounced [11,15,18]. These results have important clinical implications. Although ensitrelvir demonstrated promise in reducing antigen levels and hospitalization duration, its role in clinical management requires further exploration. Additionally, the few significant differences in fever resolution across groups suggest that symptom management should be addressed with supportive care alongside antiviral therapy.

Study limitations

The limitations of this study include the relatively small sample size and the absence of long-term follow-up data. Notably, the findings were derived primarily from hospitalized elderly patients, limiting their generalizability to pediatric and young adult populations. Additionally, the analysis was confined to only three antiviral drugs. Future research should focus on larger, diverse populations and assess long-term outcomes, including the post-acute sequelae of COVID-19.

## Conclusions

This study compared the effectiveness of remdesivir, molnupiravir, and ensitrelvir in managing hospitalized patients with COVID-19, focusing on key outcomes, including SARS-CoV-2 antigen levels, hospitalization duration, and fever resolution. While our study suggests the advantages of ensitrelvir regarding antigen clearance and reduced hospitalization duration, the overall effectiveness of these antiviral agents is similar, especially in terms of symptomatic relief. This finding underscores the need for personalized treatment approaches and further research to optimize COVID-19 management strategies.
